# Fatty Acid Composition of Hepatopancreas and Gonads in Both Sexes of Orange Mud Crab, *Scylla olivacea* Cultured at Various Water Flow Velocities

**DOI:** 10.21315/tlsr2020.31.2.5

**Published:** 2020-08-06

**Authors:** Muhammad Taufik, Ismail Shahrul, Abdul Rahman Mohd Nordin, Mhd Ikhwanuddin, Ambok Bolong Abol-Munafi

**Affiliations:** 1 Institute of Tropical Aquaculture and Fisheries, Universiti Malaysia Terengganu, 21030 Kuala Nerus, Terengganu, Malaysia; 2 Faculty of Ocean Engineering, Technology and Informatics, Universiti Malaysia Terengganu, 21030 Kuala Nerus, Terengganu, Malaysia; 3 Faculty of Informatics and Computing, Universiti Sultan Zainal Abidin, Besut Campus, 22200 Besut, Terengganu, Malaysia; 4 STU-UMT Joint Shellfish Research Laboratory, Shantou University, 515063 Guangdong, China

**Keywords:** Aquaculture, Crustacean, Portunid Crab, Fatty Acids, Water Velocity, Akuakultur, Krustasia, Ketam Portunid, Asid Lemak, Halaju Air

## Abstract

Nutritional quality of the hepatopancreas and gonads of orange portunid mud crab, *Scylla olivacea* was evaluated for each gender under four treatment of different water velocities (0, 20, 40 and 60 cm s^−1^), in terms of nutrient reserve and nutrient for reproduction. About 56 crabs were used in this study in which fatty acids composition was analysed using gas chromatography mass-spectrometry (GC-MS). For hepatopancreas analysis, monounsaturated fatty acids (MUFAs) were present in the highest fatty acids concentration, followed by polyunsaturated fatty acids (PUFAs) and, saturated fatty acids (SFAs). However, long-chain polyunsaturated fatty acids (LC-PUFAs) were displayed in low concentration in the hepatopancreas. Total fatty acid (TFAs) composition was significantly higher at moderate velocity of 20 cm s^−1^ compared to other water velocity treatments. For gonad analysis, 20 cm s^−1^ showed the highest TFA concentration of 93.34 mg g^−1^ while, the lowest concentration of 3.90 mg g^−1^ occurred at 0 cm s^−1^. There were significant differences in male and female crab’s fatty acids contents of gonads at all flow velocities challenged (*p* < 0.05). PUFAs and MUFAs were dominant while, SFAs were observed at low concentration. This study revealed that, concentration of PUFAs increased as gonad maturation increased. The decreasing concentration of hepatopancreas fatty acids over the culture period indicated that nutrient was shifted from the hepatopancreas, to be used as energy reserved to gonads for further growth of eggs and offspring. The linkages between water flow strength, hepatopancreas, and gonad fatty acids concentrations, is fundamental knowledge useful in establishing efficient habitat velocities selection which will improve aquaculture production of mud crabs with high quality broodstock.

## Abstract (ms)

Kualiti pemakanan hepatopancreas dan gonad ketam lumpur portunid jingga, *Scylla olivacea* dinilai untuk setiap jantina di bawah empat perlakuan dengan halaju air yang berbeza (0, 20, 40 dan 60 cm s^−1^), dari segi simpanan nutrien dan nutrien untuk pembiakan. Kira-kira 56 ketam digunakan dalam kajian ini di mana komposisi asid lemak dianalisis menggunakan kromatografi gas-spektometri jisim (GC-MS). Untuk analisis hepatopancreas, asid lemak tak tepu mono (MUFAs) terdapat dalam kepekatan asid lemak tertinggi, diikuti oleh asid lemak tak tepu ganda (PUFA) dan asid lemak tepu (SFA). Walau bagaimanapun, asid lemak tak tepu ganda rantai panjang (LC-PUFAs) ditunjukkan dalam kepekatan rendah pada hepatopankreas. Jumlah komposisi asid lemak (TFA) jauh lebih tinggi pada halaju sederhana 20 cm s^−1^ berbanding dengan rawatan halaju air yang lain. Untuk analisis gonad, 20 cm s^−1^ menunjukkan kepekatan TFA tertinggi 93.34 mg g^−1^ sementara, kepekatan terendah 3.90 mg g^−1^ berlaku pada 0 cm s^−1^. Terdapat perbezaan yang signifikan dalam kandungan asid lemak ketam jantan dan betina pada gonad pada semua cabaran halaju air (*p* < 0.05). PUFA dan MUFA dominan sementara, SFA diperhatikan pada kepekatan rendah. Kajian ini menunjukkan bahawa, kepekatan PUFA meningkat seiring bertambahnya kematangan gonad. Penurunan kepekatan asid lemak hepatopancreas selama tempoh kultur menunjukkan bahawa nutrien dialihkan dari hepatopancreas, untuk digunakan sebagai tenaga yang disediakan untuk gonad untuk pertumbuhan telur dan keturunan lebih lanjut. Hubungan antara kekuatan aliran air, hepatopankreas dan kepekatan asid lemak gonad, adalah pengetahuan asas yang berguna dalam mewujudkan pemilihan kecepatan habitat yang cekap yang akan meningkatkan pengeluaran akuakultur ketam lumpur dengan bahan makanan berkualiti tinggi.

**Highlights**

- Nutritional quality of the hepatopancreas and gonads of orange portunid mud crab, *Scylla olivacea* was evaluated for each gender under four treatment of different water velocities (0, 20, 40 and 60 cm s^−1^), in terms of nutrient reserve and nutrient for reproduction.
- The water velocity of 20 cm s^−1^ promotes high total fatty acid (TFAs) composition in hepatopancreas and gonad compare to other velocities.
- Fatty acid analysis on gonad revealed that more energy was accumulated for gonad maturation. It was concluded that the optimum water velocity (20 cm s^−1^) can accelerate the gonad maturation of *S. olivacea* broodstock in captivity.

## INTRODUCTION

Basic knowledge of how water velocities employed in rearing systems may impact the physiology of culture species is crucial to enhance the management of reproductive development in broodstock, especially with respect to recirculating aquaculture systems (RAS) or, indoor culture systems. The portunid crab (genus *Scylla*) is in high demand as a global aquaculture species because of its high price, unique flavour, high meat yield, rigid muscle and sweet flesh (Ikhwanuddin *et al.* 2015; Abol-Munafi & Azra 2018; Kunsook & Dumrongrojwatthana 2017; Ikhwanuddin *et al.* 2018). In portunid crab, hepatopancreas is the main tissue where fatty acids are reserved. The hepatopancreas fatty acids change when crabs mature (Abol-Munafi *et al.* 2016). The hepatopancreas also reserves energy for survival, shedding and reproduction (Sarapio et al. 2017) and, also acts as a store for vitellogenin (Wang *et al.* 2014; Liu *et al.* 2018).

Most aquatic organisms undergo long distance travel and swim for reproduction purposes, encountering exhaustion under various water flow conditions. Extensive investigation have been carried out to determine the exact capabilities of aquatic organisms to cope with this challenge including for example, determination of locomotion activities, reaction time for escaping from unfavorable conditions, maximum velocity tolerances and, energy in form of lipids reserved for long swimming and hardy migration (Hinch & Rand 1998; Hodgson & Quinn 2002; Lee *et al.* 2003; Taufik *et al.* 2019; Azra *et al.* 2019).

In natural mangrove habitats rich with food, attractive for spawning purpose are limited by water flow rates and velocities (Nagelkerken *et al.* 2008). How *S. olivacea* addresses problems posed by water velocities, whether by walking, swimming or remaining at the mercy of water current is still unclear. Investigation of this species’ capabilities to maneuver and tolerate different flow velocity conditions is necessary. The main purpose of the current study was to determine the effect that various water velocities exercise on hepatopancreas and gonad maturation in both males and females, by examining changes in fatty acids concentrations. From the mud crab activity of swimming in their actively challenging offshore migration, this investigation established the modelling hypothesis that fatty acids distribution for spawning purpose in male and female *S. olivacea* would be controlled by the velocity of water flow.

Fatty acids have been shown to have significant effect on the physiology and spawning capacity of decapod crustaceans (Taufik *et al.* 2014; 2016). Fatty acids have been suggested as the main dietary element responsible for controlling both fecundity and fertilisation of crab eggs (Alava *et al.* 2007). Research on fatty acids and their importance in aquatic reproduction and culture is common and includes study carried out by several authors on aspects such as nutritional tracing and, establishment of the role of physiological factors (Abdulkadir & Tsuchiya 2008; Taufik *et al.* 2014; Azra *et al.* 2018).

Experimentation on water flow rate effects, evaluating how velocity and duration of exposure can significantly alter the physiology of aquatic animals and affect their growth, contributes essential knowledge to aquaculture production of these species (Taufik *et al.* 2019). Environmental changes commonly cause biochemical and physiological disorder, contrary to maintenance of an ideal system (Randall & Brauner 1991). Uncertain abiotic factors such as variation in water flow can lead to changes in metabolism and, in both biochemical and morphological assimilation (Oufiero & Whitlow 2016). Higher velocities have the worst physiological impact on aquatic animals, disturbing their homeostasis and development (Hernandez *et al.* 2002).

Aquatic organisms cultured in flowing water systems have been suggested by researchers to be sensitive to fluctuating water velocity (Jobling *et al.* 1993) with high velocities influencing the physiology of some cultured aquatic species (Palstra & Planas 2011). Aquatic animals have been shown to usually move at moderate speeds under aerobic conditions (Clark *et al.* 2013) with excessive flow rates producing negative impact (Brown *et al.* 2011). The purpose of the current study is to elucidate the effect of different water velocities on *S. olivacea*, considering sex of the animal, including the linkage with hepatopancreas and gonad fatty acids analysis.

## MATERIAL AND METHOD

### Description of the Methodology

**Figure 1:**
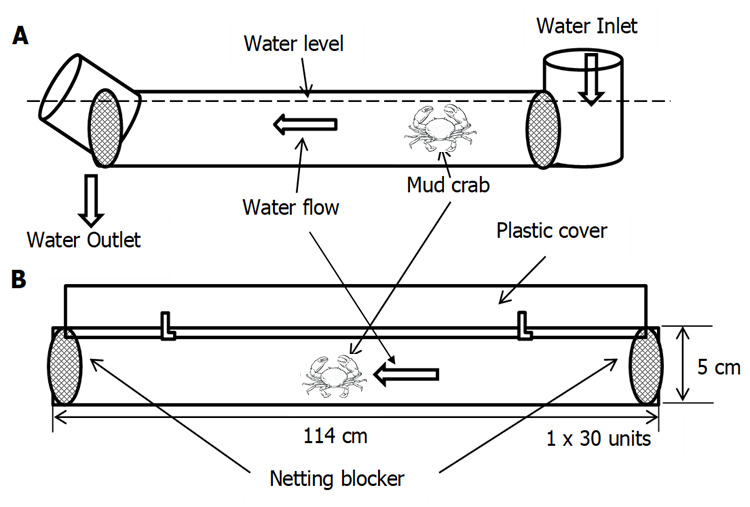
(A) Cross sectional view; (B) Top view of velocity simulator.

**Figure 2:**
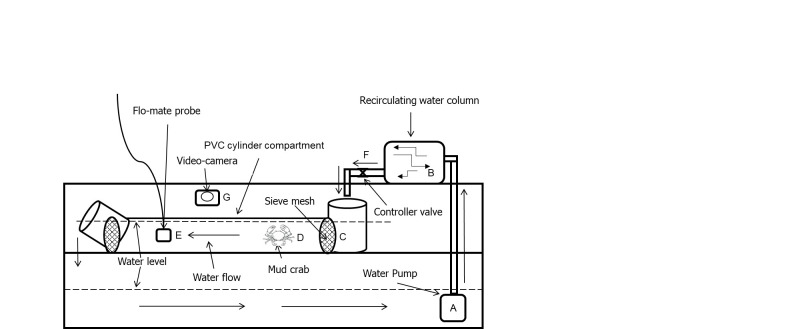
Diagrammatic representation of the water velocities apparatus used in the present study. (A) Water pump (to create artificial flow), (B) PVC cylinder compartment (containment for crabs), (C) Water inlet, (D) Water out, (E) Flo-mate probe (measure flow), (F) Control valve (manipulating flow strength), (G) Water level (actual water level in pipe and tank), (H) Mesh screens.

The crabs used in the experiments were sampled from Setiu Wetlands, Terengganu, adjacent to the Southern South China Sea, Malaysia. Immature male and female mud crabs at 50% reach maturity, with measurement carapace width of male (♂: < 8.97 cm) and female (♀: < 9.06 cm) were chosen according to the sampling method of Ikhwanuddin *et al*. (2010a; b). The samples were brought live to the hatchery of Institute of Tropical Aquaculture (AKUATROP), Universiti Malaysia Terengganu for culture. The carapace widths (CWs) were taken at the blunt point of the 9th antero-lateral spine of the carapace, scaled by vernier caliper (precision 0.01 mm). Body weight (BW) was measured by digital balance (precision 0.01 g). The crabs were acclimatised in a culture tank in normal light and ambient temperature conditions for two days before they were introduced into the treatment developed to generate velocity conditions, shown in Figs. 1 and 2. The crabs were acclimatised and experiments processed in salinity 20 ppt (Amin *et al.* 2016, 2018). The maintenance of the crabs was based on the previous published methods (Azra & Ikhwanuddin 2015, 2016; Abol-Munafi *et al.* 2017). In brief, water temperature was controlled at an average 24°C–28°C. The mud crab culture PVC pipe system and tanks were washed every day during the experiment. Washing included removal of fecal matter and, uneaten fishes and, was conducted each morning. Feeding proceeded after cleaning where raw fish (*Decapterus* spp) were given metabolic waste was managed through 50% daily exchange of water. The treatment apparatus used in this study comprised of 30 units of PVC Pipe System, two culture tanks (3000 L) and, one treated (1 tons of seawater mixed with 30 g of calcium hypochlorite and aerated 24 h under vigorous aeration before ready to use) water (20 ppt) and one holding tank (3000 L). Four different water flow velocity treatment were employed, T1, T2, T3 and T4 applying 0, 20, 40, and 60 cm s^−1^, respectively. An electric pump generated flow at 6000 L h^−1^. Inside the mangrove ecosystem, an average velocity pertains of approximately 10 cm s^−1^ (Zhang *et al.* 2012). In this study, copying natural free stream characteristic, velocities of 0, 20, 40 and 60 cm s^−1^ were generated for 60 days of specimen culture. The handy flow meter was used (Flo-Mate, model 2000, USA) which employs the Faraday method of measurement. The measurement units were assigned as centimeters per second (cm s^−1^) with accuracy of ± 5 cm s^−1^. Fig. 1 displays the PVC Pipe system conceptually in cross-section and top view and Fig. 2 displays the conceptual arrangement of the full system.

### One Step Method Procedure

After each treatment reached 0, 30 and 60 days rearing period, each of the 56 crabs were dissected. The stomach, outer carapace, gills and internal tissue, except gonads and hepatopancreas, were disposed of. The gonad and hepatopancreas were sampled and kept at −80°C for 48 h, before fatty acid (FA) extraction and esterification by the method of Abdulkadir and Tsuchiya (2008). Approximately, 200 mg to 300 mg of sample with three replicates were diluted with 4 mL of hexane, 2 mL of 14% BF_3_ in methanol and 1 mL of C19:0 solution in a 50 mL tube. A magnetic stirring bar was used as mixer and, gas space of the tubes was replaced with N_2_ over 30 s. The tube caps were closed tightly and tubes incubated for 2 h at 100°C. Upon completion, 1 mL of hexane with 2 mL H_2_O were mixed into the tubes and tubes shaken vigorously for 1 min. Subsequently, the tubes were centrifuged for 3 min at 2500 rpm (650×g). Two layers formed with the top section consisting of FAMEs. For FAME quantification, 50 mL of the FAME top layer was injected into the gas chromatograph mass spectrometer (GCMS-QP2010 Ultra). Subsequent isolation was done with a ppms-5 column (OPTIMA® 5 HT, 15 m × 0.32 mm ID, 0.25 μm film) with helium gas at pressure 50.0 kPa, velocity 0.96 mL min^−1^ and linear flow of 35.5 cm s^−1^. The total of 1 mL of FAME was inserted at 50°C for 1 min until temperature reached 300°C at 5°C min^−1^ then, the sample was held for 5 min. The concentration of fatty acids from the GCMS were recorded and analysed. In this study, student *t*-test statistical analysis was carried out using SPSS with differences shown in graph form, which were considered significant for *P* < 0.05. All data were displayed as means ± SD.

## RESULTS

**Figure 3:**
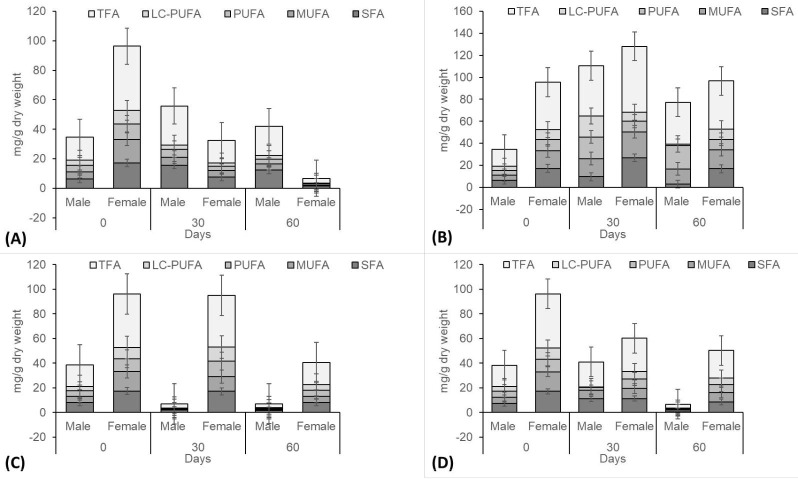
Fatty acid concentrations of hepatopancreas samples from *S. olivacea* cultured in different water velocities (A – 0 cm s^−1^; B – 20 cm s^−1^; C – 40 cm s^−1^; D – 60 cm s^−1^). * Total Fatty Acid (TFA), Long-Chain Polyunsaturated Fatty Acids (LC-PUFA), Polyunsaturated Fatty Acids (PUFA), Monounsaturated Fatty Acids (MUFA), Saturated Fatty Acids (SFA).

**Figure 4:**
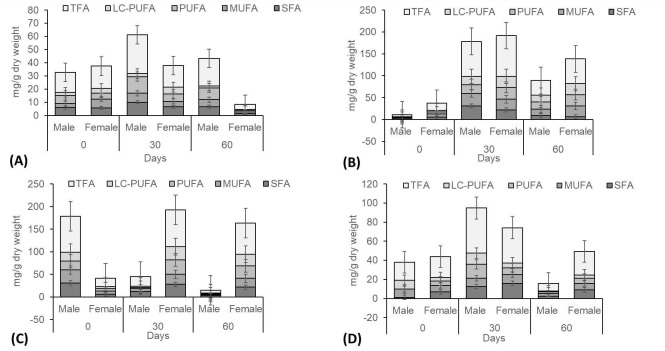
Fatty acid concentrations of gonad samples from *S. olivacea* cultured in different water velocities (A – 0 cm s^−1^; B – 20 cm s^−1^; C – 40 cm s^−1^; D – 60 cm s^−1^). * Saturated Fatty Acids (SFA), Monounsaturated Fatty Acids (MUFA), Polyunsaturated Fatty Acids (PUFA), Long-Chain Polyunsaturated Fatty Acids (LC-PUFA), Total Fatty Acid (TFA).

**Figure 5:**
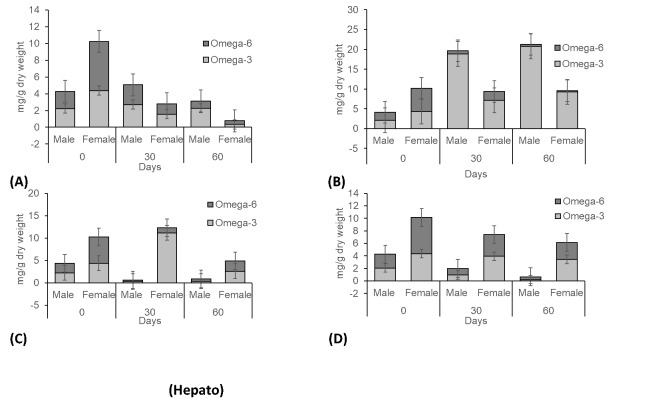
Composition of omega-3 and omega-6 profiles of hepatopancreas samples from *S. olivacea* cultured in different water velocities (A – 0 cm s^−1^; B – 20 cm s^−1^; C – 40 cm s^−1^; D – 60 cm s^−1^).

**Figure 6:**
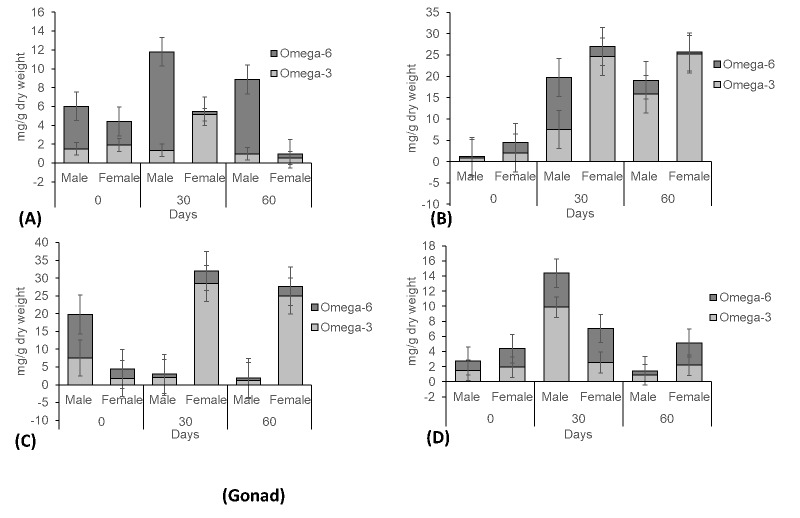
Omega-3 and omega-6 concentrations of gonad samples from *S. olivacea* cultured in different water velocities (A – 0 cm s^−1^; B – 20 cm s^−1^; C – 40 cm s^−1^; D – 60 cm s^−1^)

The average TFAs profiles of the hepatopancreas and gonad samples of *S. olivacea* among four treatments are displayed in Figs. 3 to 6. Tables 1 to 8 show the fatty acids distribution in hepatopancreas and gonads at different flow velocities. In the hepatopancreas results, LC-PUFAs was found at only a small percentage for all treatments. Velocity of 20 cm s^−1^ displayed the highest concentration of TFAs compared to other velocity treatments (Fig. 3). Fig. 3 displays that TFAs levels found in the female hepatopancreas were higher than male for all treatments. For all of the four velocities evaluated, MUFAs showed highest concentration of all fatty acids. LC-PUFAs contributed only a small proportion of the fatty acids in hepatopancreas samples (Fig. 3). Male crab showed very low hepatopancreas TFAs, which is interpreted to mean that more fatty acids are used for locomotion in males. Both male and female crabs, at velocity of 20 cm s^−1^ displayed high concentration of TFAs. Significant differences were found between male crabs in Day 30 for omega-3 and omega-6 in *S. olivacea* (Fig. 4). These results displayed that fatty acids concentration in crab hepatopancreas is diverse for culture at various water velocities.

With respect to the gonad sample results, PUFAs represented the highest fraction of all classes of fatty acids. Velocity of 20 cm s^−1^ contributed peak TFA content, compared to other treatment velocities (Fig. 5). In Fig. 5, the analysis of TFAs of male crabs (♂: 79.6 mg g^−1^) and female crabs (♀: 93.34 mg g^−1^) were the highest recorded at day 30 for 20 cm s^−1^, compared with other velocities. Male crab displayed only a small fraction of fatty acids composition at treatment velocity of 40 cm s^−1^ (Fig. 5). Higher composition fractions were found of omega-3 and omega-6 for treatment velocity of 20 cm s^−1^ in both sexes of crabs, compared to other treatments (Fig. 6). These investigations have shown that appropriate velocity in culture habitat will accelerate the gonad maturation of *S. olivacea* while, strong velocities will retard the gonad maturation process.

## DISCUSSION

Nutritional value of fatty acids is crucial to the diet of cultured species and, they exert a major impact on other vital requirements such as survival, spawning, and repair of damaged tissue (Amran *et al.* 2018). The current investigation determined the fluctuation of fatty acids inside the hepatopancreas and gonads of the target species at different water velocities. Fatty acids profiling between sexes of *S. olivacea* at different water velocities is displayed in Tables 1 to 8.

In hepatopancreas, at velocity 20 cm s^−1^, MUFAs was highest with the range of 0.70 to 35.07 mg g^−1^, SAFAs with a range of 1.30 to 16.70 mg g^−1^ and, PUFAs with a range of 0.90 to 9.27 mg g^−1^. The TFAs levels recorded by sex of crabs showed significant difference, especially at treatment velocities 40 and 60 cm s^−1^. Both PUFAs and MUFAs were found to be significantly higher (*P* < 0.05) in female crabs compared with male crabs. Teshima and Kanazawa (1983) claimed that fatty acids concentration in the hepatopancreas reduced as ovarian maturation increased and, that female crabs more quickly achieve gonadal maturation than male crabs.

About 37 types of fatty acids were found in female and male hepatopancreas of *S. olivacea*. Overall, TFA composition in samples of both sexes of this species of crab contains similar fatty acids profiles to those described by researchers for other crab species (Naczk *et al.* 2004). In hepatopancreas at velocity 20 cm s^−1^, the fatty acids which showed significant difference between the male and female crabs were C12:0, C17:0, C18:0, C24:0, C18:1n-9c, C18:2n-6c, C18:3n-6, C20:2n-6, and C20:5n-3 at velocity 20 cm s^−1^. The levels of major fatty acids in the current study: C16:0 (0.05 to 13.90 mg g^−1^), C18:0 (0.03 to 7.51 mg g^−1^), C23:0 (0.02 to 1.45 mg g^−1^), C16:1 (0.03 to 5.41 mg g^−1^), C18:1n-9c (0.03 to 10.30 mg g^−1^), C18:2n-6c (0.05 to 3.62 mg g^−1^), C20:4n-6 (ARA) (0.02 to 4.71 mg g^−1^), C20:5n-3 (EPA) (0.03 to 0.67mg g^−1^), and C22:6n-3 (DHA) (0.04 to 19.11 mg g^−1^) are similar with study by a number of earlier researchers (Wu *et al.* 2010; Amran *et al.* 2018). In hepatopancreas, C16:0 and C18:0 fatty acids function as energy sources (Gonzalez-Baro & Pollero 1988). Fatty acids C18:1n-9c and C16:1 have been shown to be dominant in ovarian gonads of *S. olivacea* in previous studies where, fatty acids C16:1 was also shown present at higher levels in female than male crabs (Gonzalez-Baro & Pollero 1988; Ghazali *et al.* 2017). Previous studies have determined that C18:2n-6c is responsible for growth and nutritional storage in *S. olivacea* (Ghazali *et al.* 2017), and, is not present with significant difference between sexes.

The variation in concentration of specific fatty acids recorded every 30 day, may have been due to the different velocities involved in study. The current work reviews specific functions for specific kinds of fatty acids in aquatic animals but, the specific role of individual fatty acids still continues to unravel. For examples, PUFAs profiles, especially omega-3 and omega-6 increased at days 0 and 30. However for days 60, the concentration of PUFAs in the hepatopancreas decreased drastically. This effect might have been the PUFAs was transferred, to tissue muscle to sustain individual position in different flow condition and, to gonads for maturation. Study by Ravid *et al.* (1999) claimed the building blocks of PUFAs were mainly constructed by triacylglycerols, phospholipid (PL) and, and cholesterols where these lipid types were importantly responsible for plasma membrane construction and, hormone sites. Omega-3 and omega-6 are the most important class of PUFAs involved in transformation of MUFAs to other forms of PUFAs (Lee *et al.* 2016). Omega-3 commonly involved in integral component in cell membrane and precursors of eicosanoids (Hurtado *et al.* 2007). Transformation enzymes serve to change linoleic acids (LA) to other forms of long chain PUFAs, such as docosahexaenoic acid (DHA) and eicosapentanoic acid (EPA), which play a vital role of increasing tissue development, that is important in aquatic animal culture and, is also essential for larval survival (Naceur *et al.* 2008).

LC-PUFAs synthesis initiate with Δ6 desaturation which insert double bond among carbon 6 and 7 of LA and α-linolenic acid (ALA) forming γ-linolenic and stearidonic acid (SDAs). Both of these fatty acids are elongated to form dihomo-γ-linolenic acid (DHGL) and eicosatetraenoic acid (ETA) by insertion two carbon through Δ6-specific elongase. Furthermore, these fatty acids are desaturase by Δ5-desaturase to form AA and EPA (Qiu 2003). Then, EPA is elongated to docosapentaenoic acid (DPA) by Δ5-elongase. Finally DPA was desaturase by Δ4-desaturase to produces DHA (Venegas-Caleron *et al.* 2010).

In this study’s consideration of mud crab gonad, TFAs concentration at treatment velocity of 20 cm s^−1^ was significantly greater than at other velocities (*P* < 0.05). The analysis of 35 (minimum) to 36 (maximum) types of fatty acids in crab gonads showed major components of 16:0, 18:0, 24:1 and 22:6n-3 (Tables 4–8). The concentrations of EPA and DHA were higher for treatment velocity of 20 cm s^−1^ (Fig. 4). DHA plays important role in fertility by mobilised from hepatopancreas to ovary for vitellogenesis and oogenesis (Tantikitti *et al*. 2015). The major component of gonadal fatty acids for both sexes were 16:0, 18:0 and 18:1n-9. These fatty acids are important nutrition for female mud crab during ovarian development (Ying *et al.* 2006) and provide migration energy to offshore for spawning. Highest PUFAs accumulated in the ovary at Day 60 for treatment velocity 20 cm s^−1^ and, was about 25.80 mg g^−1^ greater than the concentration recorded for the male testis (16.26 mg g^−1^). These results of the current study are in agreement with Ying *et al.* (2006) and Alava *et al.* (2007). It can be concluded that PUFA are crucial in gonad development. Composition of fatty acids such as 18:2n-6c, 20:4n-6, and 22:6n-3 in the ovary increased with duration of the culture period, most obviously for treatment velocity of 20 cm s^−1^. These fatty acids act as structural component of phospholipid which involved in cell membranes and lipoprotein transportation (Lytle *et al.* 1990). Previous studies have elaborated that fatty acids are fraction of PL and, the mainstay of lipoprotein transition and cell membrane structure and also, that they are crucial for prawn spawning, insemination and hatching (Lytle *et al.* 1990). The fatty acids 18: 2n-6c might be assumed to essential for embryonic growth of *Portunus pelagicus* crab (Taufik *et al.* 2016).

Under moderate velocity (20 cm s^−1^), crab were observed to swim freely and at 60 days of culture fatty acids accumulated highest in the gonads, compared with other treatment. This importantly, energy from dietary intake was allocated for gonad development rather than for locomotive activities. However, in high velocity (60 cm s^−1^), fatty acids accumulated lowest in the gonad, seemly more energy was used to tolerate in high velocity rather than used for gonad development. It is concluded that suitable habitat velocities can accelerate gonadal growth and maturation. In the current study, females were also observed to reach earlier maturation for treatment velocity of 20 cm s^−1^ than for other treatments.

This current study represents flow velocity as an additional parameter which, if appropriately selected will accelerate broodstock maturation period. The application of velocity treatment to enhance biochemical and gonad maturation in crab is not well studied, which is not the case for fish (Arbelaez-Rojas & Moraes 2013). No research has been conducted on the effect of manipulation of velocities on transfer of lipids and/or proteins, by which to enhance egg development. It has been studied for a range of aquatic organisms that exercise, brought on by habitat velocity, breaks down fatty acids accumulation (Hernandez *et al.* 2002) but, the same type of study has not been carried out for the mud crab. Habitat velocity has been shown to improve the accumulation of fatty acids in white flesh at the development stage. Female *S. olivacea*, at treatment velocity of 20 cm s^−1^, had stopped feeding and lipids reserves were decreased (Fig. 4). The results suggest that velocities trigger vitellogenesis synthesis and gonad development. Vitellogenesis, the process of formation and accumulation of yolk, is central to oogenesis and ovarian maturation. Estrogen hormones, particularly 17β-estradiol (E_2_), increase the synthesis of vitellogenin (*Vtg*) in by enhancing the transcription of the *Vtg* gene. An estrogen receptor (ER) and heat shock protein 90 (Hsp90) (Fliss *et al.* 2000; Wu & Chu 2008) are used to facilitate this process. Previous work has shown that E_2_ can stimulate the development of vitellogenin synthesis and oocyte in kuruma prawn, *Marsupenaeus Japanese* (Yano & Hoshino 2006). A research of *Metapenaeus ensis* also showed that Hsp90 played a role in controlling the synthesis of vitellogenin (Wu & Chu 2008). Therefore, further research should be concentrated on hormonal manipulation by manipulate velocity.

The novelty of this study has suggested that hepatopancreas and gonad in both sexes of *S. olivacea* were more developed at treatment velocity of 20 cm s^−1^, compared to other treatment velocities. This moderate velocity can support optimum development of the hepatopancreas. As moderate velocity stimulates good growth for crab, it should serve as suitable culture water velocity for this species. Moreover, females presented higher fatty acids content than males, with female using fatty acids for ovarian maturation, while males utilise fatty acids for seeking shelter, fighting, looking for food and, mating. Biochemical fatty acids assessment showed that the component fractions of the fatty acids, for both sexes accumulated strongly across maturation stages, in suitable water velocity of 20 cm s^−1^, compared to other treatment velocities can induced high gonad capacity. The results obtained from this work will hopefully serve as a foundation for further physiological experiments in order to establish broodstock maturation development of *S. olivacea*, especially at grow-out stage.

## Figures and Tables

**Table 1:** Fatty acids profiles (mg g^−1^ dry weight) of hepatopancreas samples from both sexes *S. olivacea* broodstock reared at different water velocities (0 cm s^−1^). Values are mean ± SD, *n* = 56.

Days	0		30		60	

Fatty acids	Male	Female	Male	Female	Male	Female
C8:0	0.36±0.03	0.15±0.05	0.09±0.04	0.45±0.05	0.03±0.01	0.08±0.03
C10:0	0.06±0.02	0.13±0.02	0.11±0.02	0.03±0.02	0.04±0.03	0.06±0.05
C12:0	0.06±0.01	0.03±0.02	0.03±0.02	0.03±0.02	0.03±0.01	0.08±0.02
C14:0	0.15±0.04	0.23±0.02	0.95±0.13	0.29±0.04	0.05±0.02	0.08±0.05
C15:0	0.20±0.03	0.36±0.04	0.33±0.06	0.20±0.05	0.15±0.04	0.08±0.06
C16:0	1.63±0.11a	4.71±0.19a	2.65±0.04a	2.63±0.06a	2.32±0.12a	0.18±0.05c
C17:0	0.58±0.03	1.31±0.19	0.26±0.03	0.25±0.05	0.13±0.02	0.04±0.03
C18:0	0.90±0.04a	3.15±0.30a	1.26±0.04a	1.76±0.06a	1.03±0.08a	0.14±0.03c
C20:0	0.15±0.05	0.39±0.04	0.55±0.04	0.20±0.05	0.35±0.04	0.06±0.05
C22:0	0.15±0.04	0.28±0.02	5.93±0.06	0.15±0.05	5.58±0.23	0.08±0.04
Total SFA	6.36	17.13	15.63	7.41	12.35	1.39
C14:1	0.24±0.03	0.07±0.03	0.07±0.02	0.05±0.04	0.03±0.01	0.04±0.02
C16:1	0.71±0.07	1.43±0.45	0.72±0.04	0.71±0.04	0.53±0.03	0.14±0.07
C17:1	0.05±0.04	0.59±0.17	0.41±0.05	0.04±0.03	0.26±0.03	0.05±0.03
C20:1	0.70±0.03	0.15±0.05	0.07±0.03	0.11±0.03	0.03±0.02	0.05±0.04
C18:1N9T	2.21±0.11	10.30±0.17	2.91±0.06	2.62±0.04	2.48±0.08	0.19±0.04
C18:1N9C	0.03±0.03	0.09±0.03	0.06±0.03	0.04±0.04	0.03±0.02	0.03±0.02
C22:1N9	0.05±0.02	0.03±0.02	0.06±0.04	0.17±0.03	0.03±0.01	0.07±0.04
Total MUFA	4.73	15.96	5.36	4.74	4.14	0.82
C18:2N6T	0.05±0.02	0.11±0.06	0.36±0.04	0.11±0.04	0.23±0.01	0.16±0.03
C18:2N6C	0.49±0.03	0.05±0.04	1.65±0.23	0.26±0.06	0.53±0.04	0.04±0.03
C18:3N6	0.09±0.01	0.60±0.03	0.13±0.05	0.20±0.02	0.03±0.02	0.07±0.06
C20:3N6	0.07±0.02	0.39±0.07	0.14±0.06	0.11±0.03	0.05±0.03	0.10±0.05
C20:4N6	1.35±0.04	4.71±0.15	0.06±0.03	0.57±0.08	0.02±0.01	0.07±0.05
n-6	2.05	5.87	2.35	1.24	0.87	0.44
C18:3N3	0.03±0.02	0.38±0.02	0.05±0.04	0.03±0.02	0.04±0.02	0.08±0.04
C20:3N3	0.12±0.02	0.07±0.02	0.06±0.03	0.15±0.03	0.03±0.01	0.09±0.04
C20:5N3	0.67±0.11	0.29±0.08	0.16±0.02	0.61±0.07	0.05±0.04	0.13±0.05
C22:6N3	1.76±0.19	3.63±0.15	2.44±0.05	0.78±0.02	2.15±0.05	0.04±0.03
n-3	2.23	4.37	2.71	1.57	2.27	0.34
C20:2	0.04±0.01	0.12±0.04	0.21±0.02	0.05±0.04	0.13±0.05	0.03±0.02
C22:2	0.06±0.02	0.16±0.04	0.19±0.04	0.04±0.02	0.05±0.02	0.14±0.05
Total PUFA	4.37	10.51	5.46	2.9	3.33	0.94
Total LC-PUFA	3.62	9.08	2.86	2.22	2.31	0.43
TFAs	15.47±0.53a	43.60±1.39a	26.45±1.44b	15.05±0.68b	19.82±0.33a	3.14±0.1.15c
(n-3)/n-6) ratio	1.09	0.74	1.17	1.27	2.61	0.8

*Notes*: Different letter showed significant different among days. C:16 and C:18 responsible for energy source (Amran *et al.* 2018).

**Table 2:** Fatty acids profiles (mg g^−1^ dry weight) of hepatopancreas samples from both sexes *S. olivacea* broodstock reared at different water velocities (20 cm s^−1^). Values are mean ± SD, *n* = 56.

Days	0		30		60	

Fatty acids	Male	Female	Male	Female	Male	Female
C8:0	0.36±0.03	0.15±0.05	0.43±0.03	0.29±0.07	0.26±0.02	0.32±0.03
C10:0	0.06±0.02	0.13±0.02	0.14±0.03	0.07±0.02	0.31±0.02	0.02±0.01
C12:0	0.09±0.04	0.03±0.02	0.04±0.04	0.04±0.02	0.18±0.04	0.03±0.02
C14:0	0.15±0.04	0.23±0.02	0.03±0.01	1.20±0.22	0.07±0.02	0.19±0.04
C15:0	0.20±0.03	0.36±0.04	0.04±0.03	0.90±0.02	0.02±0.01	0.11±0.03
C16:0	1.63±0.11a	4.71±0.19a	7.76±0.29c	12.02±1.47c	0.65±0.02d	1.53±0.06a
C17:0	0.58±0.03	1.31±0.19	0.05±0.03	1.38±0.15	0.06±0.03	0.12±0.03
C18:0	0.90±0.04a	3.15±0.30a	0.15±0.03c	5.64±0.74a	0.41±0.02c	1.25±0.06a
C20:0	0.15±0.05	0.39±0.04	0.05±0.03	0.30±0.13	0.03±0.02	0.15±0.04
C22:0	0.15±0.04	0.28±0.02	0.04±0.02	0.19±0.01	0.13±0.02	0.05±0.01
C24:0	0.02±0.01	0.06±0.02	0.07±0.04	0.26±0.17	0.09±0.04	0.04±0.01
Total SFA	6.34	17.06	9.57	26.83	2.85	4.92
C14:1	0.12±0.04	0.07±0.03	0.05±0.04	0.12±0.02	0.05±0.01	0.04±0.02
C16:1	0.71±0.07	1.43±0.45	0.12±0.03	5.04±0.59	0.18±0.02	0.40±0.03
C17:1	0.05±0.04	0.59±0.17	0.04±0.04	1.20±0.21	0.02±0.01	0.07±0.02
C20:1	0.70±0.03	0.15±0.05	0.05±0.02	1.86±0.21	0.20±0.02	0.06±0.02
C18:1N9T	2.21±0.11	0.09±0.03	10.47±0.31	0.02±0.00	0.57±0.02	0.06±0.02
C18:1N9C	0.09±0.03	10.30±0.17	0.03±0.02	12.27±1.31	0.03±0.01	1.69±0.06
C22:1N9	0.04±0.02	0.03±0.02	0.05±0.03	0.03±0.03	0.06±0.03	0.10±0.01
Total MUFA	4.66	15.96	16.51	23.67	1.84	3.14
C18:2N6C	0.49±0.03	0.05±0.04	0.08±0.03	0.62±0.13	0.05±0.01	0.15±0.07
C18:3N6	0.09±0.01	0.60±0.03	0.57±0.03	0.38±0.14	0.02±0.01	0.04±0.02
C20:3N6	0.07±0.02	0.39±0.07	0.08±0.05	0.45±0.04	0.15±0.0	0.03±0.01
C20:4N6	1.35±0.04	4.71±0.15	0.06±0.02	0.80±0.28	0.26±0.02	0.02±0.02
n-6	2	5.76	0.79	2.25	0.48	0.26
C18:3N3	0.03±0.02	0.38±0.02	0.07±0.03	0.07±0.02		
C20:3N3	0.11±0.05	0.07±0.02	0.05±0.04	0.03±0.01	0.13±0.05	0.01±0.01
C20:5N3	0.32±0.02	0.29±0.08	0.04±0.02	0.23±0.11	0.14±0.04	0.50±0.04
C22:6N3	1.76±0.05	3.63±0.15	8.69±0.21	6.60±0.50	0.43±0.02	1.77±0.03
n-3	2.1	4.37	8.85	7.12	0.77	2.28
C22:2	0.17±0.07	0.16±0.04	0.04±0.02	0.13±0.02	0.12±0.05	0.04±0.02
Total PUFA	4.49	10.28	9.71	9.51	1.37	2.57
Total LC-PUFA	3.6	9.08	8.91	8.11	1.11	2.34
TFAs	15.38±0.69a	43.29±1.36a	35.80±0.83c	60.01±7.20c	6.07±0.58d	10.64±0.68d
(n-3)/n-6) ratio	1.11	0.76	11.27	3.27	1.61	10.34

*Notes*: Different letter showed significant different among days. C:16 and C:18 responsible for energy source (Amran *et al.* 2018).

**Table 3:** Fatty acids profiles (mg g^−1^ dry weight) of hepatopancreas samples from both sexes *S. olivacea* broodstock reared at different water velocities (40 cm s^−1^). Values are mean ± SD, *n* = 56.

Days	0		30		60	

Fatty acids	Male	Female	Male	Female	Male	Female
C8:0	0.38±0.02	0.15±0.05	0.06±0.02	0.07±0.04	0.33±0.03	0.15±0.04
C10:0	0.08±0.03	0.13±0.02	0.09±0.04	0.25±0.03	0.03±0.01	0.05±0.03
C12:0	1.63±0.28	0.03±0.02	0.35±0.04	0.04±0.04	0.04±0.02	0.07±0.03
C14:0	0.17±0.02	0.23±0.02	0.05±0.03	0.66±0.02	0.06±0.01	0.34±0.03
C15:0	0.20±0.03	0.36±0.04	0.07±0.03	0.33±0.05	0.09±0.02	0.25±0.04
C16:0	1.72±0.05a	4.71±0.19a	0.11±0.02b	6.62±0.06a	0.19±0.01b	2.66±0.08a
C17:0	0.61±0.03	1.31±0.19	0.04±0.01	0.65±0.05	0.14±0.02	0.52±0.03
C18:0	0.88±0.02a	3.15±0.30a	0.07±0.02b	5.30±0.20a	0.03±0.02b	1.89±0.04a
C20:0	0.16±0.03	0.39±0.04	0.13±0.02	0.43±0.02	0.04±0.02	0.18±0.03
C22:0	0.18±0.02	0.28±0.02	0.06±0.03	0.36±0.03	0.08±0.03	0.10±0.01
Total SFA	8.27	17.31	1.88	17.11	1.57	8.17
C14:1	0.07±0.03	0.07±0.03	0.10±0.01	0.10±0.01	0.09±0.05	0.09±0.03
C16:1	0.71±0.06	1.43±0.45	0.10±0.02	1.88±0.09	0.07±0.01	0.64±0.05
C17:1	0.03±0.01	0.59±0.17	0.11±0.03	1.12±0.16	0.06±0.03	0.16±0.02
C20:1	0.72±0.05	0.15±0.05	0.04±0.01	0.87±0.04	0.07±0.04	0.55±0.04
C18:1N9T	2.18±0.02	10.30±0.17	0.12±0.02	6.52±0.07	0.20±0.02	2.17±0.05
C18:1N9C	0.08±0.04	0.09±0.03	0.08±0.03	0.15±0.03	0.03±0.01	0.06±0.03
C22:1N9	0.06±0.02	0.03±0.02	0.08±0.04	0.07±0.03	0.02±0.01	0.07±0.05
Total MUFA	4.64	15.96	0.76	12.25	0.66	5.02
C18:2N6C	0.08±0.0	0.05±0.04	0.05±0.03	0.65±0.05	0.14±0.02	0.50±0.05
C18:2N6T	0.53±0.07	0.11±0.02	0.05±0.02	0.07±0.03	0.12±0.03	0.04±0.02
C18:3N6	0.07±0.03	0.60±0.03	0.03±0.01	0.16±0.03	0.06±0.02	0.06±0.03
C20:3N6	0.08±0.02	0.39±0.07	0.05±0.02	0.16±0.04	0.17±0.06	0.07±0.02
C20:4N6	1.36±0.03	4.71±0.15	0.04±0.03	0.14±0.08	0.23±0.07	1.65±0.03
n-6	2.12	5.87	0.22	1.16	0.49	2.32
C18:3N3	0.05±0.03	0.38±0.02	0.06±0.02	0.07±0.03	0.05±0.02	0.04±0.02
C20:3N3	0.07±0.03	0.07±0.02	0.09±0.03	0.51±0.21	0.09±0.02	0.08±0.01
C20:5N3	0.37±0.06	0.29±0.08	0.12±0.03	0.23±0.08	0.11±0.02	0.07±0.02
C22:6N3	1.75±0.04	3.63±0.15	0.06±0.03	10.34±0.15	0.10±0.01	2.40±0.13
n-3	2.24	4.37	0.33	11.15	0.35	2.59
C20:2	0.09±0.04	0.06±0.03	0.06±0.05	0.06±0.02	0.15±0.02	0.09±0.03
C22:2	0.07±0.02	0.16±0.04	0.08±0.04	0.22±0.16	0.17±0.08	0.06±0.03
Total PUFA	4.52	10.28	0.68	12.54	1.05	4.98
Total LC-PUFA	3.62	9.08	0.36	11.38	0.57	4.28
TFAs	17.43±1.13a	43.54±1.41a	3.32±0.28b	41.90±0.91a	3.28±0.13b	18.11±0.16c
(n-3)/n-6) ratio	1.05	0.76	1.49	9.71	0.72	1.12

*Notes*: Different letter showed significant different among days. C:16 and C:18 responsible for energy source (Amran *et al.* 2018).

**Table 4:** Fatty acids profiles (mg g^−1^ dry weight) of hepatopancreas samples from both sexes *S. olivacea* broodstock reared at different water velocities (60 cm s^−1^). Values are mean ± SD, *n* = 56.

Days	0		30		60	

Fatty acids	Male	Female	Male	Female	Male	Female
C8:0	0.38±0.02	0.15±0.05	0.12±0.03	0.10±0.06	0.04±0.02	0.04±0.03
C10:0	0.08±0.02	0.13±0.02	0.05±0.03	0.40±0.04	0.03±0.01	0.29±0.02
C12:0	0.08±0.04	0.03±0.02	0.18±0.03	0.20±0.05	0.03±0.01	0.07±0.01
C14:0	0.21±0.07	0.23±0.02	0.24±0.05	0.33±0.05	0.04±0.03	0.16±0.04
C15:0	0.19±0.02	0.36±0.04	0.18±0.05	0.23±0.05	0.07±0.04	0.11±0.03
C16:0	1.71±0.04a	4.71±0.19a	3.22±0.06a	3.88±0.09a	0.13±0.04b	3.34±0.09a
C17:0	0.62±0.04	1.31±0.19	0.54±0.04	0.48±0.04	0.08±0.03	0.36±0.03
C18:0	0.88±0.02a	3.15±0.30a	2.68±0.08c	2.20±0.11a	0.09±0.04b	1.84±0.25a
C20:0	0.17±0.04	0.39±0.04	0.18±0.04	0.33±0.05	0.05±0.03	0.14±0.03
C22:0	0.20±0.04	0.28±0.02	0.26±0.03	0.27±0.06	0.09±0.03	0.13±0.03
Total SFA	7.29	17.06	10.99	11.17	1.33	8.47
C14:1	0.49±0.06	0.07±0.03	0.22±0.04	0.15±0.04	0.08±0.03	0.04±0.02
C16:1	0.70±0.05	1.43±0.45	0.55±0.05	0.81±0.07	0.14±0.05	0.63±0.07
C17:1	0.05±0.03	0.59±0.17	0.36±0.04	0.24±0.04	0.05±0.02	0.13±0.03
C20:1	0.75±0.11	0.15±0.05	0.05±0.04	0.19±0.05	0.05±0.03	0.08±0.03
C18:1N9T	2.26±0.15	10.30±0.17	2.71±0.03	5.82±0.07	0.12±0.03	6.19±0.64
C18:1N9C	0.08±0.02	0.09±0.03	0.13±0.05	0.06±0.04	0.09±0.02	0.03±0.01
C22:1N9	0.07±0.03	0.03±0.02	0.25±0.04	0.09±0.04	0.4±0.03	0.04±0.02
Total MUFA	5.16	15.96	7.12	8.22	0.96	7.66
C18:2N6C	0.50±0.05	0.05±0.02	0.39±0.04	0.21±0.03	0.06±0.05	0.05±0.02
C18:2N6T	0.50±0.06	0.05±0.04	0.26±0.03	0.16±0.05	0.13±0.03	0.08±0.02
C18:3N6	0.10±0.02	0.60±0.03	0.20±0.05	0.37±0.07	0.04±0.03	0.20±0.02
C20:3N6	0.12±0.08	0.39±0.07	0.16±0.03	0.14±0.04	0.11±0.02	0.05±0.02
C20:4N6	1.47±0.13	4.71±0.15	0.04±0.02	2.58±0.05	0.11±0.02	2.33±0.03
n-6	2.2	5.8	1.04	3.47	0.45	2.71
C18:3N3	0.04±0.01	0.38±0.02	0.59±0.03	0.53±0.07	0.05±0.03	0.32±0.02
C20:3N3	0.08±0.03	0.07±0.02	0.08±0.03	0.10±0.04	0.08±0.04	0.02±0.02
C20:5N3	0.36±0.06	0.29±0.08	0.15±0.04	0.09±0.04	0.03±0.02	0.03±0.02
C22:6N3	1.67±0.15	3.63±0.15	0.15±0.03	3.22±0.07	0.06±0.04	3.07±0.05
n-3	2.07	4.37	0.96	3.94	0.22	3.45
C20:2	0.13±0.04	0.06±0.02	0.03±0.02	0.12±0.04	0.06±0.03	0.04±0.02
C22:2	0.16±0.04	0.16±0.04	0.08±0.04	0.11±0.05	0.09±0.05	0.05±0.02
Total PUFA	4.8	10.28	2.12	7.65	0.82	6.26
Total LC-PUFA	3.7	9.08	0.57	6.14	0.39	5.51
TFAs	17.32±1.29a	43.96.±1.46a	20.23±1.23a	27.04±1.29c	3.11±0.53b	22.39±0.76c
(n-3)/n-6) ratio	0.92	0.75	0.93	1.14	0.47	1.27

*Notes*: Different letter showed significant different among days. C:16 and C:18 responsible for energy source (Amran *et al.* 2018).

**Table 5:** Fatty acids profiles (mg g^−1^ dry weight) of testis samples from testis and ovary *S. olivacea* broodstock reared at different water velocities (0 cm s^−1^). Values are mean ± SD, *n* = 56.

Days	0		30		60	

Fatty acids	Male	Female	Male	Female	Male	Female
C8:0	0.14±0.01	0.53±0.02	0.41±0.05	0.26±0.03	0.27±0.03	0.12±0.03
C10:0	0.10±0.01	0.03±0.02	0.16±0.04	0.03±0.02	0.07±0.04	0.04±0.03
C12:0	0.06±0.03	0.07±0.02	0.06±0.01	0.08±0.03	0.06±0.03	0.07±0.03
C14:0	0.55±0.03	0.10±0.01	0.10±0.02	0.09±0.01	0.06±0.02	0.10±0.04
C15:0	0.56±0.03	0.12±0.03	0.27±0.05	0.05±0.01	0.13±0.02	0.08±0.03
C16:0	0.16±0.05a	1.30±0.06a	0.82±0.04c	1.88±0.03a	0.04±0.02d	0.14±0.04b
C17:0	0.35±0.13	0.22±0.01	0.10±0.04	0.19±0.02	0.04±0.01	0.06±0.03
C18:0	0.69±0.01a	0.99±0.09a	0.92±0.05a	2.01±0.08a	0.66±0.04a	0.12±0.03b
C20:0	0.80±0.07	0.02±0.02	0.76±0.04	0.15±0.04	0.53±0.02	0.06±0.02
C22:0	0.61±0.03	0.22±0.03	1.61±0.18	0.16±0.02	1.34±0.10	0.04±0.01
Total SFA	5.98	5.71	10.04	6.5	6.58	1.29
C14:1	0.19±0.18	0.02±0.02	0.31±0.05	0.09±0.01	0.20±0.01	0.05±0.03
C16:1	0.51±0.42	0.71±0.02	0.30±0.02	0.36±0.02	0.22±0.02	0.07±0.02
C17:1	0.08±0.01	0.13±0.02	0.10±0.03	0.07±0.02	0.05±0.03	0.10±0.04
C20:1	0.37±0.17	0.51±0.02	0.37±0.05	0.08±0.01	0.21±0.02	0.08±0.06
C18:1N9T	0.28±0.17	1.56±0.05	1.19±0.14	2.39±0.01	0.91±0.03	0.18±0.04
C18:1N9C	0.06±0.02	0.01±0.00	0.10±0.05	0.08±0.03	0.03±0.01	0.06±0.03
C22:1N9	0.04±0.02	0.15±0.03	0.26±0.02	0.17±0.02	0.14±0.04	0.08±0.04
Total MUFA	2.9	6.72	6.79	4.1	5.33	1.41
C18:2N6C	0.04±0.02	0.09±0.02	0.26±0.05	0.03±0.02	0.11±0.02	0.12±0.03
C18:2N6T	0.25±0.16	0.69±0.06	0.11±0.06	0.18±0.02	0.03±0.01	0.07±0.02
C18:3N6	0.19±0.02	0.02±0.01	1.19±0.05	0.06±0.03	0.67±0.50	0.05±0.03
C20:3N6	0.49±0.05	0.03±0.01	0.18±0.05	0.06±0.03	0.05±0.01	0.09±0.06
C20:4N6	0.26±0.24	1.66±0.04	0.84±0.04	0.03±0.02	0.62±0.04	0.12±0.04
n-6	4.5	2.5	10.92	0.36	7.88	0.45
C18:3N3	0.05±0.01	0.09±0.03	0.17±0.05	0.12±0.03	0.05±0.03	0.13±0.06
C20:3N3	0.05±0.04	0.02±0.01	0.11±0.04	0.07±0.02	0.06±0.03	0.08±0.04
C20:5N3	0.67±0.11	0.28±0.03	0.87±0.04	0.07±0.03	0.66±0.04	0.12±0.09
C22:6N3	0.74±0.19	1.53±0.02	0.36±0.02	4.87±0.03	0.20±0.02	0.19±0.04
n-3	1.51	1.91	1.51	5.13	0.98	0.53
C22:2	0.32±0.21	0.02±0.01	0.21±0.02	0.09±0.03	0.13±0.02	0.13±0.02
Total PUFA	6.33	4.52	12.65	5.71	8.99	1.2
Total LC-PUFA	2.21	3.51	2.37	5.1	1.59	0.61
TFAs	15.21±2.63a	16.95±0.28a	29.47±2.06c	16.32±0.71a	20.90±0.78c	3.90±0.15c
(n-3)/n-6) ratio	0.34	0.76	0.14	15.17	0.12	1.17

*Notes*: Different letter showed significant different among days. C:16 and C:18 responsible for energy source (Amran *et al.* 2018).

**Table 6:** Fatty acids profiles (mg g^−1^ dry weight) of testis samples from male *S. olivacea* broodstock reared at different water velocities (20 cm s^−1^). Values are mean ± SD, *n* = 56.

Days	0		30		60	

Fatty acids	Male	Female	Male	Female	Male	Female
C8:0	0.35±0.04	0.53±0.02	0.14±0.01	0.05±0.01	0.12±0.03	0.22±0.03
C10:0	0.11±0.04	0.03±0.02	0.10±0.01	0.07±0.02	0.22±0.04	0.01±0.01
C14:0	0.04±0.03	0.10±0.01	0.55±0.03	0.45±0.06	0.10±0.02	0.06±0.03
C15:0	0.06±0.02	0.12±0.03	0.56±0.03	0.30±0.02	0.05±0.04	0.07±0.02
C16:0	0.19±0.03a	1.30±0.06a	9.16±0.05b	9.63±0.26c	6.65±0.20b	1.67±0.02a
C17:0	0.02±0.01	0.22±0.01	2.35±0.13	1.27±0.06	0.07±0.02	0.23±0.06
C18:0	0.16±0.02a	0.99±0.09a	5.69±0.01b	7.84±0.18c	0.12±0.08a	2.26±0.23a
C20:0	0.15±0.03	0.02±0.02	0.80±0.07	0.25±0.04	0.33±0.04	0.11±0.03
C22:0	0.07±0.01	0.22±0.03	0.61±0.03	0.61±0.04	0.25±0.03	0.15±0.04
Total SFA	2.35	5.66	30.98	22.24	9.52	6.72
C14:1	0.03±0.02	0.01±0.01	0.19±0.18	0.11±0.03	0.44±0.04	0.09±0.01
C16:1	0.06±0.02	0.71±0.02	2.18±0.29	3.54±0.25	0.21±0.04	0.44±0.03
C17:1	0.12±0.02	0.13±0.02	0.08±0.01	1.62±0.79	0.55±0.04	0.15±0.04
C20:1	0.03±0.01	0.51±0.02	0.37±0.17	1.12±0.01	0.65±0.04	0.10±0.03
C18:1N9T	0.18±0.02	1.56±0.05	18.28±0.17	12.88±0.31	0.06±0.03	2.17±0.12
C18:1N9C	0.08±0.03	0.01±0.00	0.06±0.02	0.04±0.01	0.24±0.04	0.13±0.02
C22:1N9	0.14±0.03	0.15±0.03	0.04±0.02	0.03±0.01	0.23±0.05	0.09±0.04
Total MUFA	1.31	6.71	28.56	44	6.65	24.14
C18:2N6C	0.03±0.01	0.69±0.06	0.04±0.02	0.92±0.01	2.55±0.13	0.28±0.04
C18:2N6T	0.03±0.01	0.05±0.03	0.25±0.16	0.05±0.01	0.19±0.08	0.02±0.02
C18:3N6	0.03±0.02	0.02±0.01	0.19±0.02	0.26±0.13	0.09±0.02	0.02±0.03
C20:3N6	0.07±0.03	0.03±0.01	0.49±0.05	0.12±0.01	0.04±0.01	0.06±0.03
C20:4N6	0.21±0.03	1.66±0.04	11.26±0.24	1.04±0.79	0.40±0.04	0.02±0.02
n-6	0.37	2.46	12.23	2.39	3.27	0.5
C18:3N3	0.08±0.03	0.08±0.04	0.05±0.01	0.65±0.02	0.26±0.03	0.03±0.02
C20:3N3	0.14±0.03	0.02±0.01	0.05±0.04	0.15±0.04	0.24±0.04	0.11±0.03
C20:5N3	0.40±0.05	0.28±0.03	0.67±0.11	0.23±0.05	0.12±0.03	0.15±0.04
C22:6N3	0.18±0.02	1.53±0.02	6.74±0.19	23.46±0.21	5.15±0.04	4.83±0.13
n-3	0.8	2.04	7.51	24.61	5.77	5.19
C20:2	0.07±0.02	0.12±0.05	0.06±0.03	0.05±0.04	0.11±0.04	0.09±0.08
C22:2	0.09±0.04	0.02±0.01	0.32±0.21	0.04±0.01	0.11±0.03	0.03±0.01
Total PUFA	1.26	4.64	20.06	27.09	9.27	5.8
Total LC-PUFA	1	3.51	19.21	24.99	5.95	5.17
TFAs	4.93±0.67a	17.02±0.32a	79.60±2.43c	93.34±2.23c	25.44±1.29e	36.66±1.19b
(n-3)/n-6) ratio	2.18	0.83	0.61	10.99	1.77	10.58

*Notes*: Different letter showed significant different among days. C:16 and C:18 responsible for energy source (Amran *et al.* 2018).

**Table 7:** Fatty acids profiles (mg g^−1^ dry weight) of testis samples from testis and ovary *S. olivacea* broodstock reared at different water velocities (40 cm s^−1^). Values are mean ± SD, *n* = 56.

Days	0		30		60	

Fatty acids	Male	Female	Male	Female	Male	Female
C8:0	0.14±0.01	0.56±0.02	0.06±0.03	0.20±0.01	0.21±0.02	0.13±0.03
C10:0	0.10±0.01	0.06±0.04	0.08±0.04	0.18±0.04	0.04±0.01	0.07±0.03
C12:0	0.06±0.03	0.15±0.04	0.07±0.03	0.06±0.04	0.05±0.03	0.04±0.02
C14:0	0.55±0.03	0.10±0.02	0.08±0.04	0.44±0.02	0.09±0.02	0.25±0.03
C15:0	0.56±0.03	0.13±0.02	0.34±0.05	0.40±0.01	0.09±0.04	0.25±0.04
C16:0	9.16±0.05a	1.35±0.03a	0.20±0.01b	10.88±0.03c	0.20±0.02b	8.43±0.40c
C17:0	2.35±0.13	0.25±0.04	1.96±0.06	1.40±0.01	1.17±0.15	1.16±0.07
C18:0	5.69±0.01a	1.01±0.17a	0.17±0.02b	9.18±0.05b	0.10±0.05b	7.43±0.45b
C20:0	0.80±0.07	0.03±0.02	0.10±0.01	0.62±0.03	0.07±0.02	0.46±0.03
C22:0	0.61±0.03	0.22±0.03	0.46±0.06	0.27±0.01	0.20±0.01	0.15±0.02
Total SFA	31.03	6.24	11.95	27.77	2.88	21.46
C14:1	0.19±0.18	0.07±0.03	0.90±0.08	0.24±0.03	0.14±0.03	0.13±0.02
C16:1	2.18±0.29	0.73±0.06	0.57±0.02	3.16±0.05	0.04±0.02	2.57±0.16
C17:1	0.08±0.01	0.15±0.05	0.11±0.02	0.64±0.03	0.08±0.03	0.47±0.03
C20:1	0.37±0.17	0.53±0.02	0.49±0.02	1.31±0.02	0.31±0.02	0.86±0.03
C18:1N9T	18.28±0.17	1.44±0.21	0.28±0.02	12,92±0.03	0.04±0.01	12.55±0.13
C18:1N9C	0.06±0.02	0.03±0.01	0.52±0.08	0.06±0.02	0.18±0.03	0.04±0.02
C22:1N9	0.04±0.02	0.16±0.05	0.22±0.01	0.07±0.04	0.08±0.04	0.04±0.03
Total MUFA	28.56	6.8	6.17	21.99	1.75	19.5
C18:2N6C	0.04±0.02	0.69±0.07	0.05±0.03	1.57±0.06	0.05±0.04	1.35±0.10
C18:2N6T	0.25±0.16	0.25±0.02	0.16±0.02	0.24±0.02	0.02±0.01	0.13±0.02
C18:3N6	0.19±0.02	0.03±0.03	0.13±0.02	0.75±0.05	0.27±0.04	0.58±0.03
C20:3N6	0.49±0.05	0.08±0.03	0.45±0.06	0.71±0.03	0.21±0.02	0.53±0.07
C20:4N6	11.26±0.24	1.6±0.09	0.21±0.02	0.24±0.07	0.18±0.03	0.15±0.03
n-6	12.23	2.65	1	3.51	0.73	2.73
C20:3N3	0.05±0.04	0.03±0.01	0.05±0.03	0.06±0.04	0.03±0.01	0.04±0.02
C20:5N3	0.67±0.11	1.26±0.04	0.63±0.02	1.05±0.03	0.03±0.02	1.05±0.02
C22:6N3	6.74±0.19	1.51±0.08	1.10±0.01	27.38±0.03	1.04±0.03	23.88±0.36
n-3	7.51	1.8	2.02	28.49	1.23	24.97
C20:2	0.04±0.02	0.07±0.04	0.06±0.03	0.02±0.02	0.09±0.02	0.06±0.02
C22:2	0.15±0.03	0.03±0.01	0.14±0.02	0.11±0.04	0.10±0.02	0.05±0.02
Total PUFA	19.88	5.51	3.16	32.14	2.16	27.81
Total LC-PUFA	19.21	4.48	2.43	29.45	1.5	25.65
TFAs	79.48±2.27a	18.55±0.49a	21.27±0.22b	81.45±0.47c	6.78±0.20c	68.76±0.81d
(n-3)/n-6) ratio	0.61	1.06	2.03	8.11	1.69	9.15

*Notes*: Different letter showed significant different among days. C:16 and C:18 responsible for energy source (Amran *et al*. 2018).

**Table 8:** Fatty acids profiles (mg g^−1^ dry weight) of testis samples from testis and ovary *S. olivacea* broodstock reared at different water velocities (60 cm s^−1^). Values are mean ± SD, *n* = 56.

Days	0		30		60	

Fatty acids	Male	Female	Male	Female	Male	Female
C8:0	0.14±0.01	0.56±0.03	0.10±0.02	2.53±0.37	0.12±0.03	0.16±0.04
C10:0	0.10±0.01	0.03±0.02	0.28±0.04	0.15±0.03	0.17±0.06	0.06±0.03
C12:0	0.06±0.03	0.24±0.03	0.02±0.01	0.47±0.03	0.10±0.02	0.27±0.03
C14:0	0.55±0.03	0.12±0.03	0.34±0.04	0.25±0.04	0.07±0.04	0.14±0.03
C15:0	0.56±0.03	0.15±0.04	0.18±0.02	0.37±0.03	0.04±0.02	0.21±0.02
C16:0	9.16±0.05a	1.37±0.07a	3.76±0.12c	1.68±0.10b	0.09±0.02e	1.27±0.16c
C17:0	2.35±0.13	0.25±0.04	0.68±0.12	0.39±0.05	0.07±0.02	0.26±0.04
C18:0	5.69±0.01a	1.04±0.14a	2.92±0.07a	1.44±0.10c	0.12±0.08b	1.09±0.06a
C20:0	0.80±0.07	0.03±0.01	0.29±0.01	0.20±0.01	0.08±0.04	0.08±0.04
C22:0	0.61±0.03	0.25±0.05	0.32±0.05	0.40±0.02	0.17±0.05	0.25±0.04
Total SFA	31.14	6.75	12.5	15.87	2.13	8.98
C14:1	0.19±0.18	0.03±0.02	0.18±0.02	0.15±0.04	0.32±0.08	0.06±0.03
C16:1	2.18±0.29	0.74±0.04	0.73±0.06	0.86±0.03	0.24±0.04	0.52±0.16
C17:1	0.08±0.01	0.18±0.02	2.05±0.01	0.32±0.03	0.17±0.05	0.15±0.03
C20:1	0.37±0.17	0.55±0.02	0.13±0.02	0.75±0.02	0.61±0.05	0.40±0.06
C18:1N9T	18.28±0.17	1.62±0.06	3.18±0.01	1.90±0.04	0.34±0.05	1.55±0.07
C18:1N9C	0.06±0.02	0.03±0.01	0.08±0.04	0.16±0.03	0.19±0.02	0.07±0.02
C22:1N9	0.04±0.02	0.15±0.04	0.12±0.07	0.36±0.03	0.22±0.03	0.25±0.04
Total MUFA	28.56	7.05	8.68	8.95	3.28	6.83
C18:2N6C	0.04±0.02	0.67±0.04	0.65±0.01	0.88±0.04	0.07±0.04	0.65±0.03
C18:2N6T	0.25±0.16	0.67±0.05	0.06±0.02	0.84±0.08	0.19±0.02	0.64±0.04
C18:3N6	0.19±0.02	0.05±0.01	0.05±0.03	0.28±0.04	0.12±0.03	0.19±0.04
C20:3N6	0.49±0.05	0.06±0.02	0.08±0.04	0.24±0.06	0.15±0.03	
C20:4N6	11.26±0.24	1.66±0.03	3.66±0.03	2.26±0.18	0.07±0.06	1.31±0.29
n-6	12.23	2.44	4.49	4.5	0.53	2.94
C20:3N3	0.05±0.04	0.05±0.03	0.21±0.04	0.16±0.04	0.21±0.03	0.08±0.03
C20:5N3	0.67±0.11	0.31±0.07	1.08±0.02	0.59±0.07	0.38±0.15	0.45±0.09
C22:6N3	6.74±0.19	1.57±0.08	7.10±0.00	1.80±0.07	0.18±0.03	1.66±0.12
n-3	7.51	1.93	9.89	2.55	0.92	2.19
C22:2	0.32±0.21	0.05±0.02	0.05±0.02	0.15±0.03	0.06±0.03	0.08±0.03
Total PUFA	20.06	4.41	14.47	7.2	1.58	5.21
Total LC-PUFA	19.21	3.64	11.75	5.05	0.92	3.65
Total Fatty Acids	79.53±2.40a	18.21±1.08a	35.66±0.81c	32.02±1.35c	6.99±0.25d	21.02±0.37b
(n-3)/n-6) ratio	0.61	0.79	2.2	0.57	1.76	0.75

*Notes*: Different letter showed significant different among days. C:16 and C:18 responsible for energy source (Amran *et al.* 2018).

## References

[b1-tlsr-31-2-79] Abdulkadir S, Tsuchiya M (2008). One-step method for quantitative and qualitative analysis of fatty acids in marine animal samples. Journal of Experimental Marine Biology and Ecology.

[b2-tlsr-31-2-79] Abol-Munafi AB, Azra MN (2018). Climate change and the crab aquaculture industry: problems and challenges. Journal of Sustainability Science and Management.

[b3-tlsr-31-2-79] Abol-Munafi AB, Mukrim MS, Amin RM, Azra MN, Azmie G, Ikhwanuddin M (2016). Histological profile and fatty acid composition in hepatopancreas of blue swimming crab, *Portunus pelagicus* (Linnaeus, 1758) at different ovarian maturation stages. Turkish Journal of Fisheries and Aquatic Sciences.

[b4-tlsr-31-2-79] Abol-Munafi AB, Pilus N, Amin RM, Azra MN, Ikhwanuddin M (2017). Digestive enzyme profiles from foregut contents of blue swimming crab, *Portunus pelagicus* from Straits of Johor, Malaysia. Journal of the Association of Arab Universities for Basic and Applied Sciences.

[b5-tlsr-31-2-79] Alava VR, Quinitio ET, Pedro JB, Priolo FM, Orozco ZG, Wille M (2007). Lipid and fatty acids in wild and pond-reared mud crab *Scylla serrata* during ovarian maturation and spawning. Aquaculture Research.

[b6-tlsr-31-2-79] Amin-Safwan A, Muhd-Farouk H, Mardhiyyah MP, Nadirah M, Ikhwanuddin M (2018). Does water salinity affect the level of 17β-estradiol and ovarian physiology of orange mud crab, *Scylla olivacea* (Herbst, 1796) in captivity?. Journal of King Saud University Science.

[b7-tlsr-31-2-79] Amin-Safwan A, Muhd-Farouk H, Nadirah M, Ikhwanuddin M (2016). Effect of water salinity on the external morphology of ovarian maturation stages of orange mud crab, *Scylla olivacea* (Herbst, 1796) in captivity. Pakistan Journal of Biological Sciences.

[b8-tlsr-31-2-79] Amran A, Ariffin MH, Noordin NM, Ikhwanuddin M (2018). Morphological, biochemical and histological analysis of mud crab ovary and hepatopancreas at different stages of development. Animal Reproduction Science.

[b9-tlsr-31-2-79] Arbelaez-Rojas GA, Moraes G (2013). Effect of water velocity on intermediary metabolism of juvenile matrinxã fish (*Brycon amazonicus*). Revista Colombiana de Ciencias Pecuarias.

[b10-tlsr-31-2-79] Azra MN, Ikhwanuddin M (2015). Larval culture and rearing techniques of commercially important crab, Portunus pelagicus (Linnaeus, 1758): Present status and future prospects. Songklanakarin Journal of Science and Technology.

[b11-tlsr-31-2-79] Azra MN, Ikhwanuddin M (2016). A review of maturation diets for mud crab genus Scylla broodstock: present research, problems and future perspective. Saudi Journal of Biological Sciences.

[b12-tlsr-31-2-79] Azra MN, Chen JC, Ikhwanuddin M, Abol-Munafi AB (2018). Thermal tolerance and locomotor activity of blue swimmer crab *Portunus pelagicus* instar reared at different temperatures. Journal Thermal Biology.

[b13-tlsr-31-2-79] Azra MN, Ikhwanuddin M, Abol-Munafi AB (2019). Behavioural data on instar crab movement at different thermal acclimation. Data in Brief.

[b14-tlsr-31-2-79] Brown EJ, Bruce M, Pether S, Herbert NA (2011). Do swimming fish always grow fast? investigating the magnitude and physiological basis of exercise-induced growth in juvenile New Zealand yellowtail king fish, *Seriola lalandi*. Fish Physiology Biochemistry.

[b15-tlsr-31-2-79] Clark TD, Sandblom E, Jutfelt F (2013). Aerobic scope measurements of fishes in an era of climate change: respirometry, relevance and recommendations. Journal of Experimental Biology.

[b16-tlsr-31-2-79] Fliss AE, Benzeno S, Rao J, Caplan AJ (2000). Control of estrogen receptor ligand binding by Hsp90. Journal of Steroid Biochemistry and Molecular Biology.

[b17-tlsr-31-2-79] Ghazali A, Azra MN, Noordiyana MN, Abol-Munafi AB, Ikwanuddin M (2017). Ovarian morphological development and fatty acids profile of mud crab (*Scylla olivacea*) fed with various diets. Aquaculture.

[b18-tlsr-31-2-79] Gonzalez-Baro MDR, Pollero RJ (1988). Lipid characterization and distribution among tissues of the freshwater crustacean *Macrobrachium borellii* during an annual cycle. Comparative Biochemistry Physiology-B: Comparative Biochemistry.

[b19-tlsr-31-2-79] Hernandez MD, Mendiola P, Costa J, Zamora S (2002). Effects of intensive exercise on rainbow trout growth, body composition and metabolic responses. Journal of Physiology and Biochemistry.

[b20-tlsr-31-2-79] Hinch SG, Rand PS (1998). Swim speeds and energy use of upriver-migrating sockeye salmon (*Oncorhynchus nerka*): role of local environment and fish characteristics. Canadian Journal of Fisheries and Aquatic Sciences.

[b21-tlsr-31-2-79] Hodgson S, Quinn TP (2002). The timing of adult sockeye salmon migrations into fresh water: adaptations by populations to prevailing thermal regimes. Canadian Journal of Zoology.

[b22-tlsr-31-2-79] Hurtado MA, Racotta IS, Civera R, Ibarra L, Hernandez-Rodriguez M, Palacios E (2007). Effect of hypo- and hypersaline conditions on osmolality and Na^+^/K^+^-ATPase activity in juvenile shrimp (*Litopenaeus vannamei*) fed low- and high-HUFA diets. Comparative Biochemistry and Physiology Part A: Molecular & Integrative Physiology.

[b23-tlsr-31-2-79] Ikhwanuddin M, Bachok Z, Hilmi MG, Azmie G, Zakaria MZ (2010a). Species diversity, carapace width-body weight relationship, size distribution and sex ratio of mud crab, genus *Scylla* from Setiu Wetlands of Terengganu coastal waters, Malaysia. Journal of Sustainability Science and Management.

[b24-tlsr-31-2-79] Ikhwanuddin M, Bachok Z, Mohd-Faizal WWY, Azmie G, Abol-Munafi AB (2010b). Size of maturity of mud crab *Scylla olivacea* (Herbst, 1796) from mangrove areas of Terengganu coastal waters. Journal of Sustainability Science and Management.

[b25-tlsr-31-2-79] Ikhwanuddin M, Lan SS, Abdul Hamid N, Fatihah Zakaria SN, Azra MN, Siti Aisah A, Abol-Munafi AB (2015). The embryonic development of orange mud crab, *Scylla olivacea* (Herbst, 1796) held in the captivity. Iranian Journal of Fisheries Sciences.

[b26-tlsr-31-2-79] Ikhwanuddin M, Azmie G, Nahar SF, Wee W, Azra MN, Abol-Munafi AB (2018). Testis maturation stages of mud crab (*Scylla olivacea*) broodstock on different diets. Sains Malaysiana.

[b27-tlsr-31-2-79] Jobling M, Baarvik BM, Christiansen JS, Jorgensen EH (1993). The effects of prolonged exercise training on growth performance and production parameters in fish. Aquaculture International.

[b28-tlsr-31-2-79] Kunsook C, Dumrongrojwatthana P (2017). Species diversity and abundance of marine crabs (portunidae: decapoda) from a collapsible crab trap fishery at Kung Krabaen Bay, Chanthaburi Province, Thailand. Tropical Life Sciences Research.

[b29-tlsr-31-2-79] Lee CG, Farrell AP, Lotto A, MacNutt MJ, Hinch SG, Healey MC (2003). The effect of temperature on swimming performance and oxygen consumption in adult sockeye (*Oncorhynchus nerka*) and coho (*O. kisutch*) salmon stocks. Journal of Experimental Biology.

[b30-tlsr-31-2-79] Lee JM, Lee H, Kang S, Park WJ (2016). Fatty acid desaturases, polyunsaturated fatty acid regulation, and biotechnological advances. Nutrients.

[b31-tlsr-31-2-79] Liu M, Pan J, Liu Z, Cheng Y, Gong J, Wu X (2018). Effect of estradiol on vitellogenesis and oocyte development of female swimming crab, *Portunus trituberculatus*. Aquaculture.

[b32-tlsr-31-2-79] Lytle JS, Lytle TF, Ogle LT (1990). Polyunsaturated fatty acid profiles as comparative tool in assessing maturation diets of *Penaeus vannamei*. Aquaculture.

[b33-tlsr-31-2-79] Naceur HB, Jenhani ABR, El- Cafsi M, Romdhane MS (2008). Determination of biological characteristics of *Artemia salina* (Crustacea: Anostraca) population from Sabkhet Sijoumi (NE Tunisia). Transitional Waters Bulletin.

[b34-tlsr-31-2-79] Naczk M, Williams J, Brennan K, Liyanapathirana C, Shahidi F (2004). Compositional characteristics of green crab (*Carcinus maenas*). Food Chemistry.

[b35-tlsr-31-2-79] Nagelkerken I, Blaber SJM, Bouillon S, Green P, Haywood M, Kirton LG, Meynecke JO (2008). The habitat function of mangroves for terrestrial and marine fauna: a review. Aquatic Botany.

[b36-tlsr-31-2-79] Oufiero CE, Whitlow KR (2016). The evolution of phenotypic plasticity in fish swimming. Current Zoology.

[b37-tlsr-31-2-79] Palstra AP, Planas JV (2011). Fish under exercise. Journal of Fishery Physiological and Biochemical.

[b38-tlsr-31-2-79] Qiu X (2003). Biosynthesis of docosahexaenoic acid (DHA, 22:6–4, 7, 10, 13, 16, 19): two distinct pathways. Prostaglandins, Leukotrienes and Essential Fatty Acids.

[b39-tlsr-31-2-79] Randall D, Brauner C (1991). Effects of environmental factors on exercise in fish. Journal of Experimental Biology.

[b40-tlsr-31-2-79] Ravid T, Tietz A, Khayat M, Boehm E, Michelis R, Lubzens E (1999). Lipid accumulation in the ovaries of a marine shrimp *Penaeus semisulcatus* (de Haan). Journal of Experimental Biology.

[b41-tlsr-31-2-79] Sarapio E, Santos JT, Model JFA, De Frage LS, Vinagre AS, Martins TL, Da Silva RSM, Trapp M (2017). Glycerogenesis in the hepatopancreas of the crab Neohelice granulat: diet, starvation and season effects. Journal of Comparative Biochemical and Physiologycal B.

[b42-tlsr-31-2-79] Tantikitti C, Konoona R, Pongmaneerat J (2015). Fatty acid profiles and carotenoids accumulation in hepatopancreas and ovary of wild female mud crab (*Scylla paramamosain*, Estampador, 1949). Songklanakarin Journal of Sciences and Technology.

[b43-tlsr-31-2-79] Taufik M, Bachok Z, Azra MN, Ikhwanuddin M (2014). Identification and determination of the fatty acid composition of *Portunus pelagicus* in Setiu Wetland Areas, Terengganu, Malaysia by GC-MS. Middle-East Journal of Scientific Research.

[b44-tlsr-31-2-79] Taufik M, Bachok Z, Azra MN, Ikhwanuddin M (2016). Effects of various microalgae on fatty acid composition and survival rate of the blue swimming crab *Portunus pelagicus* larvae. Indian Journal of Geo Marine Sciences.

[b45-tlsr-31-2-79] Taufik M, Shahrul I, Ikhwanuddin M, Ambok Bolong AM (2019). Experimental data on behavioral, hepato-gonado-somatic indexes and total lipid of mud crab, *Scylla olivacea* at different velocity levels. Data in Brief.

[b46-tlsr-31-2-79] Teshima S, Kanazawa A (1983). Variation in lipid compositions during the ovarian maturation of the prawn (*Penaeus japonicas*). Journal of Bulletin Japanese Chemistry Social Science Fishery.

[b47-tlsr-31-2-79] Venegas-Caleron M, Sayanova O, Napier JA (2010). An alternative to fish oils: metabolic engineering of oil-seed crops to produce omega-3 long chain polyunsaturated fatty acids. Progress in Lipid Research.

[b48-tlsr-31-2-79] Wang W, Wu X, Liu Z, Zheng H, Cheng Y (2014). Insights into hepatopancreatic functions for nutrition metabolism and ovarian development in the crab *Portunus trituberculatus*: Gene discovery in the comparative transcriptome of different hepatopancreas stages. PLoS One.

[b49-tlsr-31-2-79] Wu X, Zhou B, Cheng Y, Zeng C, Wang C, Feng L (2010). Comparison of gender differences in biochemical composition and nutritional value of various edible parts of the blue swimmer crab. Journal of Food Composition Analysis.

[b50-tlsr-31-2-79] Wu LT, Chu KH (2008). Characterization of heat shock protein 90 in the shrimp *Metapenaeus ensis*: Evidence for its role in the regulation of vitellogenin synthesis. Molecular Reproduction and Development.

[b51-tlsr-31-2-79] Yano I, Hoshino R (2006). Effects of 17 beta-estradiol on the vitellogenin synthesis and oocyte development in the ovary of kuruma prawn (*Marsupenaeus japonicus*). Comparative Biochemistry and Physiology - Part A: Molecular & Integrative Physiology.

[b52-tlsr-31-2-79] Ying XP, Yang WX, Zhang YP (2006). Comparative studies on fatty acid composition of the ovaries and hepatopancreas at different physiological stages of the Chinese mitten crab. Aquaculture.

[b53-tlsr-31-2-79] Zhang K, Liu H, Li Y, Xu H, Shen J, Rhome J, Smith TJ (2012). The role of mangroves in attenuating storm surges. Estuarine Coastal and Shelf Science.

